# Applications of Chondrocyte-Based Cartilage Engineering: An Overview

**DOI:** 10.1155/2016/1879837

**Published:** 2016-08-18

**Authors:** Abdul-Rehman Phull, Seong-Hui Eo, Qamar Abbas, Madiha Ahmed, Song Ja Kim

**Affiliations:** ^1^Department of Biological Sciences, College of Natural Sciences, Kongju National University, Gongjudaehakro 56, Gongju 32588, Republic of Korea; ^2^Department of Pharmacy, Quaid-i-Azam University, Islamabad 45320, Pakistan

## Abstract

Chondrocytes are the exclusive cells residing in cartilage and maintain the functionality of cartilage tissue. Series of biocomponents such as different growth factors, cytokines, and transcriptional factors regulate the mesenchymal stem cells (MSCs) differentiation to chondrocytes. The number of chondrocytes and dedifferentiation are the key limitations in subsequent clinical application of the chondrocytes. Different culture methods are being developed to overcome such issues. Using tissue engineering and cell based approaches, chondrocytes offer prominent therapeutic option specifically in orthopedics for cartilage repair and to treat ailments such as tracheal defects, facial reconstruction, and urinary incontinence. Matrix-assisted autologous chondrocyte transplantation/implantation is an improved version of traditional autologous chondrocyte transplantation (ACT) method. An increasing number of studies show the clinical significance of this technique for the chondral lesions treatment. Literature survey was carried out to address clinical and functional findings by using various ACT procedures. The current study was conducted to study the pharmacological significance and biomedical application of chondrocytes. Furthermore, it is inferred from the present study that long term follow-up studies are required to evaluate the potential of these methods and specific positive outcomes.

## 1. Introduction

The chondrocytes are the only cells found in cartilage. These are unique in their secluded nature, having no direct access to the vascular system. The chondrocytes are providing mechanical support as a key functional component and permit smooth pain-free articulation in cartilage. Chondrocytes demonstrate distinctive features such as being metabolically active to maintain the turnover of extracellular matrix (ECM) by synthesising glycoprotein, collagens, proteoglycans, and hyaluronan. Chondrocytes have higher matrix to cell volume occupying 10% of tissue volume and can be correlated with functional feature of mammalian articular cartilages [1]. Protein and gene expression, metabolic activity, and surface markers are common sharing features of the chondrocytes and differences can be observed along the depth of the cartilage tissues. Various studies described the chondrocytes as mechanocyte, capable of responding to the mechanical signals in connective tissue lineage [2].

**Figure 1:**
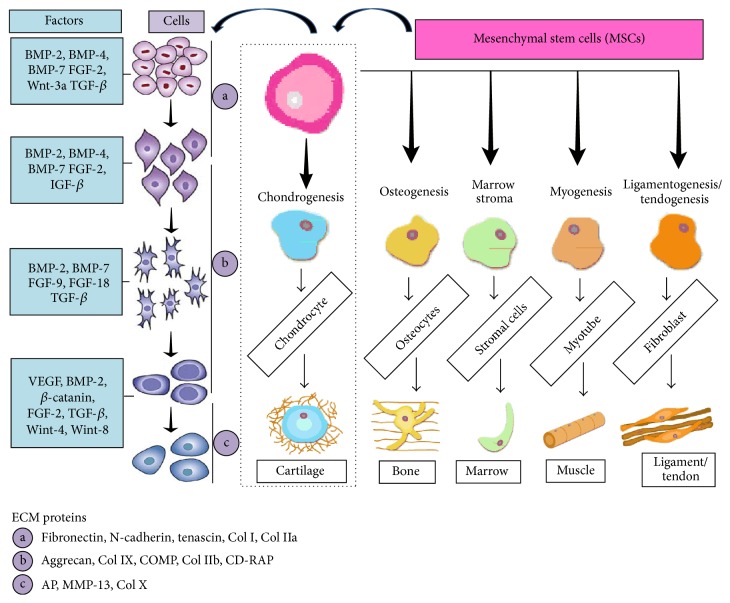
Mesenchymal stem cells (MSCs) differentiations towards chondrocytes and other cell types. Differentiation and growth factors profile are schematically represented in sequence. Characteristic extracellular matrix (ECM) proteins at different stages are presented. Col, collagen; COMP, cartilage oligomeric protein; CD-RAP, cartilage-derived retinoic acid-sensitive protein; AP, alkaline phosphatase; MMP, matrix metalloprotease; BMPs, bone morphogenetic proteins; FGF, fibroblast growth factor; Wnt, Wingless Factors; TGF, transforming growth factor; IGF, insulin-like growth factor; VEGF, vascular endothelial growth factor.

Cellular condensation is the initial marker of differentiation which occurs during chondrogenesis and the formation of skeletal elements. Mesenchymal stem cells (MSCs) are the multipotent cells arising from lateral plate mesoderm, cranial neural crest, and somites. Most of the molecular events involved in the differentiation of the MSCs towards chondrocytes are yet to be explored. Committed progenitor cell sequentially differentiated as chondroprogenitor cell, chondroblasts, chondrocytes, and finally hypertrophic chondrocytes. Sequential events of differentiation are shown in Figure 1, while a number of signalling components are required for the inductions of chondrogenesis which have been identified and further understanding of downstream regulation is in progress. Here we summarize the factors which act as commencing agents in chondrogenesis. The Sox9 transcription factor is the key regulator of chondrogenesis, which is expressed during condensation of mesenchymal progenitor cells and results in the generation of spherical immature chondrocytes containing cartilage primordial [3]. Chondrocytes within cartilage primordial continue to express Sox9 and then undergo maturation. About twentyfold increase in volume of the cells takes place in this process [4] and resultant cells are called hypertrophic chondrocytes.

Usually mature chondrocytes are round or polygonal with flattened edges in their structure but also found to have discoid or flattened shape (Figure 1). The chondrocyte cells are normally found in lacunae (matrix cavities) and establishing 5–10% of cartilage volume. These cells are about 13 mm diameter, playing a fundamental role in the maintenance of the ECM stabilization [5]. The mature chondrocytes have the abundant Golgi apparatus and rough endoplasmic reticulum and possess prominent nucleus. Under higher magnification human chondrocytes appeared to have oval or round nucleoli and a pair of centrioles in a juxtanuclear cell centre in electron micrograph. Further, occasional lipid droplets, elongated mitochondria, enlarged Golgi region, and basophilic cytoplasm are found in regenerating cartilage or new forming matrix [6]. The pericellular matrix is present around these cells and chondrocytes lack cell-to-cell interactions. Chondrocytes undergo phenotypic variation depending on the conditions of the growth environment. Therefore, these cells show loss in phenotype state when grown in monolayer cultures. The variations in the shape are the consequence of the several signalling pathways, matrix-specific components formation, and gene expression. Additionally, compressive load modifies the cellular expression through mechanotransduction phenomenon [1]. Location and origin determine the fate of chondrocytes. The cell in epiphyseal growth plates leads to hypertrophy and terminal differentiation assists in ossification of endochondral tissues. Hypertrophic chondrocytes in calcified cartilaginous matrix facilitate the bone setting on it. In this perspective, chondrocytes undergo apoptotic cell death or metaplasia or transdifferentiate to osteoblast resulting in the conversion of the cartilage to bone [7]. Factors involved in the fate of chondrocytes are yet to be interpreted.

Chondrocytes are low proliferative in their nature, having vital role in homeostasis by regulating and producing ECM components. The primary function of the chondrocytes is to provide structural support to articular, nasal, and tracheal cartilage that is required in tissue functions and withstands physical deformation. The chondrocytes play dynamic role in growth epiphyseal plate through involvement of different mechanisms such as an increase in matrix secretions, cell volume during terminal differentiation (hypertrophy), and proliferation of the cells. These mechanisms contribute to growth but may vary between joints, growth period, and also epiphyseal growth plate as supporting diaphysis and epiphysis. Due to the absence of the vascular system in articular cartilage, chondrocytes rely on the diffusion for metabolite exchange to get nourishment. Chondrocytes metabolism usually takes place in the range of >1–10% oxygen tension depending on the location of the cell. Therefore, most of the energy is acquired through glycolysis. Various genes are upregulated when chondrocytes are cultured at low oxygen tension which includes connective tissue growth factor and TGF-*β* [8], whereas adoption at lower oxygen tension elevates AP-1 and HIF transcription levels [1]. Chondrocytes maintain the metabolism of the ECM matrix, which results in stabilization of the cartilage structure and cellular environment. The aggrecan which is the aggregate of the chondroitin sulphate and type II collagen provides resistance against compression. Type II collagen gives tensile strength to cartilage [9]. Interlinking of type II collagen with other collagens leads to the formation of endoskeleton [10]. Large aggregate of the keratin sulphate glycosaminoglycan (GAG) chains and aggrecan monomers associate with hyaluronic acid (HA) and link proteins [11]. The negative charge of the aggrecans makes them suitable for drawing the water in order to resist the compression. Along with aggrecan type II collagen and other minor constituents, including cartilage oligomeric matrix protein, decorin, biglycan, and type VI, IX, and XI collagen play important role in controlling organization and the matrix structure [12]. Joint biomechanics is purely dependent on the structural integrity and composition of the ECM which is performed by chondrocytes. The arthritis is the foremost degenerative pathology related to the chondrocytes. Arthritis can be noninflammatory as osteoarthritis or inflammatory as rheumatoid arthritis. These days much attention is given to identify the genes involved in abnormal development of skeletal structures and to explore the potential therapeutic option. The cartilage destruction is the shared feature found in both classes of pathologies. The matrix metalloproteinases (MMPs), disintegrin, and metalloproteinase with a thrombospondin motif (ADAM-TS4 and ADAM-TS5) are responsible for cartilage destruction through cleaving collagen and proteoglycan moieties of the extracellular matrix. Excessive loading causes ECM degradation which is the result of the loss of water content and pericellular matrix volume [6]. Comprehending the physiopathological transduction mechanisms of chondrocyte cells in the presence of mechanical signals is well-meaning. During physiological conditions, normal load is involved in ECM maintenance by inducing the synthesis of aggrecan. The chondrocytes are also important for bone growth in epiphyseal growth plates through ossification. Present work describes the significance of various factors in the development of chondrocytes and is to speculate on the biomedical importance of these cells in different fields.

The articular cartilage pathology may be driven by cytokines, growth factors, variations in biomechanics, and cellular responses [13]. Autologous chondrocyte transplantation (ACT), abrasive chondroplasty, microdrilling/microfracturing, and osteochondral grafting are extensively executed procedures by the orthopedic surgeons for the articular cartilage repair. Among other experimental approaches, tissue engineering is promising option for the structural and functional reconstruction of the cartilage tissues. Cells such as chondrocytes, signalling molecules, and matrix scaffold are the three fundamental constituents of such an approach [14]. Development of regenerative cartilage medicine has been emerging since 1980s with the first case of autologous chondrocytes transplantation. However, injuries and other complications, including the source of chondrocyte and dedifferentiation, are the major questions to be solved. Progressive advancement in chondrocyte-based therapy is reported [15], and initially during 1997 FDA approved the autologous cultured chondrocyte (Carticel®: Genzyme Biosurgery, Cambridge, MA, USA) for the repair of cartilage defects [16].

## 2. Focal Cells for Cartilage Repair

Cell based therapy is a biological therapy, involving the use of cells into tissues to treat degenerative or age related disorders and it is rapidly growing since the last 3-4 decades for the treatment of cartilage related defects. Autologous transplantation/implantation of chondrocytes (ACT/ACI), intra-articular injection of meniscus stem/progenitor cells, and autologous matrix induced chondrogenesis represent the current methods for cartilage repairing through cell based therapies [17, 18], although cell therapy is an established part of the health care systems and exponentially growing with the evolving of these systems. However, combinatorial approaches of using cell therapy with tissue engineering and biomaterials are increasing these days.

### 2.1. Chondrocyte

Chondrocytes are the exclusive cells in articular cartilage, having size of 10–13 *µ*m diameters and involved in the synthesis of the cellular matrix constituents [19]. Due to the absence of vascular, nervous, and lymphatic systems in articular cartilage, chondrocyte cells survive in an anaerobic environment and get nutrients from the synovial fluid through diffusion. Phenotypic and metabolic profile of the chondrocytes varies in different zones within cartilage. Chondrocytes are widely used in cartilage tissue engineering, though different questions are there to be answered, including dedifferentiation during* in vitro* expansion, to manage demand of chondrocyte quantity. Assuming the accessibility of articular cartilage by surgery, native chondrocytes offer logical perspective for cartilage repair.* Ex vivo* culturing of the chondrocytes was first attempted in 1970s and reduced production of type II collagen and proteoglycans were reported upon expansion in monolayer culturing [20]. This process has been known as dedifferentiation. Currently, a procedure for optimization of* ex vivo* selection and expansion of chondrocytes is an active research area in the field of tissue engineering. In 2001, the idea of chondrocyte quality control was introduced and it was suggested that enriched stable population of chondrocytes could be used for more reproducible results of autologous chondrocyte implantation [21]. Furthermore, the positive association of different markers such as FGFR-3, COL2A1, and BMP-2 with stable chondrocyte phenotype was recognized. In the first autologous chondrocyte implantation clinical trial anchorage-independent growth and the expression of type II collagen were assessed to substantiate the expansion of chondrocytes [22]. These markers were found ineffective in predicting the capacity of expanded cells to produce stable cartilage tissue. Articular cartilage possesses four zones of different structures as calcified, deep, middle, and superficial. These zones offer another research area of using chondrocytes of specific zone to regenerate biomimetic functional cartilage tissue [23] and cell of each zone shows different features [24]. In addition, xenogeneic and allogeneic chondrocyte are readily available, widely studied alternative chondrocytes cell sources. However, these cells can be involved in diseases transmission and induction of immune responses. Therefore, more studies are needed to alleviate such issues in the field of xenogeneic and allogeneic chondrocytes [25]. Though there are some issues such as donor-site morbidity caused by cartilage harvest,* in vitro* chondrocyte dedifferentiation, limited cells available, and multiple surgical procedures involved, chondrocytes cell source along with biocompatible scaffolds and growth factors has been attempted in regenerative medicine for treating cartilage related defects.

### 2.2. Mesenchymal Stem Cells (MSCs)

MSCs are the important cells sources in tissue engineering approaches. These cells have higher proliferation rate and chondrodifferentiation capacity and are easy to collect from their respective tissue, such as adipose tissue, synovial membrane, and bone marrow [26, 27]. Allogeneic MSCs were reported to improve the articular cartilage quality in osteoarthritis patients compared to control which was investigated through magnetic resonance imaging (T2 mapping). MSCs cells are reported to exhibit promising results showing the potential as a new cell source for cartilage repairing techniques [28]. Implanted stem cells release growth factors, bioactive lipids, microvesicles, and cytokines having angiopoietic and anti-inflammatory effects. Probably pain relieving effects are due to paracrine effects of injected MSCs. These secreted biologicals could be used as therapeutic agents. The two promising adult MSCs cell types are adipose tissue-derived MSCs and bone marrow-derived MSCs, which are involved in the homeostatic regulation of tissues.

#### 2.2.1. Adipose Tissue-Derived MSCs (AD-MSCs)

Recently, AD-MSCs were used for treating osteoarthritis [29, 30] and it was proposed that the use of AD-MSCs is advantageous over BM-MSCs due to lower risks of complications. Intra-articular injection of cells obtained infrapatellar fat pad derived cells along with platelet-rich plasma containing several kinds of growth factors (PRP) which were used in osteoarthritis patients. Improved clinical outcomes have been reported in 2-year follow-up study and significant beneficial effects have been observed in patients treated with MSC along with PRP. [29]. Improvement in the cartilage was confirmed through magnetic resonance imaging (MRI) investigation. AD-MSCs had an advantage over BM-MSCs as obtaining cells from bone marrow is difficult and painful.

#### 2.2.2. Bone Marrow-Derived MSCs (BM-MSCs)

BM-MSCs are one of the important stem cell options in tissue engineering and different studies have reported potential of these cells for the treatment of the osteoarthritis. One of the preliminary investigations has reported the use of BM-MSCs to treat the four patients of moderate-to-severe knee osteoarthritis [16, 31]. Reduction in pain produced during the walk was reported in one-year follow-up study, while pain on a visual analogue scale was improved in all patients and overall encouraging results were observed. In another study, Orozco et al. [32] reported the MSC therapy on 12 patients; in this trial, patients were treated with more BM-MSCs comparative to previous report and diagnosed with Kellgren and Lawrence grades II to IV knee osteoarthritis. After one-year follow-up study, significantly improved cartilage quality was observed in 11 patients as detected by T2 mapping quantification. In general, preliminary studies on MSC based therapy were found effective in reducing pain. Furthermore, large scale and long term follow-up studies are required before application of MSCs therapy in clinical translation [16]; obtaining cells which is difficult and painful and risks of complications are the major disadvantages of using BM-MSCs.

### 2.3. Induced Pluripotent Stem Cells (iPSCs)

*In vitro* differentiation potential of iPSCs into cartilage makes them promising cells for chondrogenic application in different field including the clinical field. Comparatively, human iPSC pellets or combined forms of alginate hydrogel-iPSC have shown better quality of repaired cartilage tissue in animal model when treated with alginate hydrogel alone [33, 34]. Recently, iPSCs were produced by reprogramming the synovial cells obtained from osteoarthritis patients and used in the generation of the chondrocytes [35]. Although promising result has been reported, still there are various queries including optimal practices and chondrogenic efficacy of isolated cells.* Ex vivo* purification of these calls, genetic modification associated with reprogramming protocols, teratogenesis perspective, and* in vivo* tissue malformations are yet to be resolved. Furthermore, the approvals of the iPSC-based therapies for treating cartilage degeneration remain to be answered [36].

### 2.4. Embryonic Stem Cells (ESCs)

ESCs have unlimited proliferation potential and capability to differentiate into any type of somatic cell, which encourages the use of these cells in tissue engineering purposes. The research on these cells is controversial due to the use of developing embryo for the isolation of these cells. Recently,* in vitro* and* in vivo* studies indicated the chondrogenic differentiation potential in response of growth factors or coculturing with other cells as embryonic limb bud cells and chondrocytes. Various physical, diffusible factors induce chondrogenesis in aggregates of ESCs [37]. The status and application of these cells for cartilage tissue engineering are in the growing stages and various issues such as host immune-rejection and teratoma formation are yet to be resolved for adopting ESCs [37, 38]. In conclusion, these hurdles need be solved by more detailed investigations of biomedical application potential of these cells. Advantage and disadvantages of using different cell sources in tissue engineering are presented in Table 1.

## 3. Growth Factors in Chondrocyte Development, Cartilage Maintenance, and Repair

Diverse differentiation and growth factors are involved in the development of chondrocytes from MSCs, chondrocyte morphology maintenance, and cartilage formation [39]. These factors regulate the specific differentiation pathways and maintain the cartilage homeostasis. Herein, we are discussing the importance of the growth factors in cartilage synthesis and understanding the molecular mechanisms. There are different classes of factors including transforming growth factor-b (TGF-b), fibroblast growth factors (FGFs), insulin-like growth factor- (IGF-) 1, and Wingless Factors (Wnt).

### 3.1. The Transforming Growth Factor-b (TGF-b)

TGF-b factors are superclass of polypeptides and contain different factors, including TGF-b, Inhibins, activins, and bone morphogenetic proteins (BMPs). Depending on ligand, these components interact with type I and type II receptors on the cell surface and initiate the signalling cascade [40]. Upon ligand binding, activation of type I receptor occurs via type II receptor causing activation of BMP, TGF-b, and activin-binding. Downstream phosphorylation of mediators smads 1, 5, and 8 and smads 2 and 3 takes place. Nuclear translocations of smad 4 associated with phosphorylated smads are involved in the transcription functions [40].

#### 3.1.1. TGF-b

There are five isoforms of the TGF-b (TGF-b1–b5), peptides in their nature, acting as multifunctional components mostly formed in cartilage and bone. These molecules are found in most of the body cells. The presence of the TGF-b1 is limited in hypertrophic and proliferative regions of the cartilages, while highest expression of TGF-b2 is found in the hypertrophic, mineralizing zone of chondrocytes and in human long bone associated cartilages [41]. The TGF-b3 expression is also apparent in similar zones. The TGF-1 supports the chondrogenesis by inducing the differentiation of MSCs. TGF-b1 stimulation improved the expression of type II collagen, a differentiation marker in the C3H10T1/2 cells (pluripotent mesenchymal cell line). Generally, TGF-b1, TGF-b2, and TGF-b3 are effective stimulator of type II collagen and proteoglycans in chondrocytes and involved in differentiation of MSCs [42]. The TGF-b isoforms expression pattern in chick is inconsistent with expression in human cartilage. One of the previous studies on chick model showed the critical role of TGF-b in the late-stage differentiation of chondrocytes which may involve the osteogenesis [6]. Previously the supportive role of TGF-b1 ectopic cartilage formation from the MSCs was reported. It also enhances the integration of chondrocytes in endogenous tissues and helps in repairing of the cartilage defect [43]. At the same time, the different side effects are reported as TGF-b-expressing adenoviruses or TGF-b injection resulting swelling, synovial hyperplasia, and osteophyte formation [44], indicating the important role of TGF-b. Therefore, strict regulation of TGF-b is required for chondrogenesis. Moreover, TGF-b1 treatment in MSCs causes 100% chondrogenic differentiation and 25% in control cells (marrow cell) [45]. Additionally, TGF-b3 is having an important role in chondrogenic maturation in micromass pellet culture [26].

#### 3.1.2. BMPs

Bone morphogenetic proteins (BMPs) are a subgroup of the TGF-b family consisting of about 20 different members. These are also multifunctional polypeptides having an important role in chondrogenesis [46], by promoting several aspects such as terminal differentiation [47]. During* in vitro* culturing BMPs promote the upregulation of aggrecan and type II collagen expressions [48]. Furthermore, during the initial stages of chondrocyte formation BMPs induce the expression of the N-cadherin which promotes cell-cell interaction [49], required for Sox expression during chondrogenesis [50]. Type II collagen and Sox-9 expressions inducing effects have been reported in monopotential chondroprogenitor (MC615) and multipotent mesenchymal (C3H10T1/2) cells [51]. Plasmid DNA for BMP-2 expression in combination with type 1 collagen [52] and BMP-7 have shown promising healing effects by improving full-thickness cartilage defects in a rabbit model [53]; another study reported that BMP-4 induced chondrogenesis and cartilage repair in rats [54]. Moreover, BMP signalling pathway controls the differentiation of chondrocytes [55] and these proteins enhance type X collagen promoter activity results in expression of type X collagen (chondrocyte hypertrophic marker) [56]. Although introduction of BMPs in ectopic localization may be involved in osteogenesis, consequently it must be regulated for use in tissue engineering approach. Some BMPs (BMP-2, BMP-4, and BMP-7) have critical importance in cartilage repair and have been approved for clinical application [47], but their repairing potential must be validated in human.

#### 3.1.3. Cartilage-Derived Morphogenetic Proteins

One more important subcategory of TGF-b is cartilage-derived morphogenetic proteins (CDMPs) which are believed to function in chondrogenesis. CDMP-1, CDMP-2, and CDMP-3 constitute this group and are also known as growth/differentiation factor-5, factor-6, and factor-7, respectively [57]. CDMPs expression occurs during mesenchymal cell condensation to cartilaginous cores of the growing bone. Combined treatment of CDMP-1 and TGF-b1 induces the MSC to chondrocyte differentiation processes [58].* In vivo* and* in vitro* studies showed that CDMP-1 stimulates GAG and aggrecan synthesis [59]. Likewise, it seems that CDMP-2 involved terminal differentiation of chondrocytes, proteoglycan synthesis, and endochondral bone ossification in a chondrocytic cell line [57].

#### 3.1.4. Inhibins and Activins

Inhibins and activins are closely related proteins interacting with structurally related serine/threonine kinase receptors and primarily known for anterior pituitary regulation [60]. The Inhibins and activins protein complexes are the members of the TGF-b group of growth factors/pleiotropic hormones and involved in a variety of biological functions such as differentiation, erythropoiesis, liver proliferation, bone formation, angiogenesis, and functions of numerous cells [61]. One of the previous studies suggests that both these proteins directly affect the chondrocyte metabolism during chondrogenesis and osteogenesis which might be involved in bone modelling. Inhibin beta A function as progrowth signalling molecule in chondrocytes hypertrophy [62]. Inhibin and activins are involved in different pathophysiological conditions. Understanding of the mechanisms of these factors in cartilage related pathologies and the regulation is not fully explored.

### 3.2. Fibroblast Growth Factor (FGF)

There are 22 different structurally related proteins constituting the FGF group in vertebrates. Most of these proteins are secreted, excluding FGF1, FGF-2, FGF-11, FGF-12, FGF-13, and FGF-14 and all these proteins are capable of attaching to four FGF receptors (FGFRs) [63]. Numerous studies have showed the importance of FGFs in chondrocyte proliferation [64–66] and cell division. DNA and RNA synthesis stimulation are key features of these mitogens [64]. The FGFs role in ECM synthesis and inducing effects on rabbit costal chondrocyte confluence has been reported [65]. FGF interactions with specific receptors activate different signalling pathways. Ras-mitogen activated protein kinase pathways include prominent pathways as phosphoinositide-3-OH kinase-protein kinase B, phospholipase C, p38 kinase, extracellular-related kinase 1 and kinase 2, and c-Jun N-terminal kinase [67]. Mutations in FGFR coding genes can cause deregulation in skeletal development which is associated with several types of dysplasia. Using different cell models, diverse effects of the FGF types have been illustrated on chondrocyte proliferation. FGF-1, FGF-2, and FGF-18 exhibit more stimulating effects on proliferation of human growth plate chondrocytes comparative to FGF-4 and FGF-9 [68]. Similarly, FGF-2, FGF-4, and FGF-9 strongly stimulated the proliferation of avian chondrocyte, while lower degrees of stimulation have been found against FGF-6 and FGF-8 [69]. Lower numbers of studies have reported the chondrogenic effect of FGFs. The expression of the FGF receptor (FGFR3) in MSC cells (murine C3H10T1/2) causes differentiation of the chondrocytes [70], and expression of FGF18 (ligand of FGFR3) induces the production of cartilage matrix in limb bud mesenchymal cell differentiation [70]. Another study reported the possible role of the FGF-18 in cartilage repair [71]. Lower doses of the bFGF cause the downregulation of the alkaline phosphatase and increased deposition of calcium in higher alkaline producing hypertrophic chondrocytes [72]. These effects indicate the ossification and terminal chondrocytes differentiation inhibitory role of bFGF. FGF2 is the most important one among other members of the FGF class with respect to its proliferation and differentiation inducing effect in adult chondrocytes [73]. In addition, FGF-2 restored the cartilage in the articular cartilage defects of rabbit model [74]. Inconsistency and contradiction in results indicate the tightly controlled mechanism of FGF. Therefore to explore detailed functions of FGF is needed.

### 3.3. Insulin-Like Growth Factor (IGF)

IGF-1 and IGF-2 (ligands), IGF-1R and IGF-2R (receptors), IGF-binding proteases, and IGF-binding proteins collectively regulate the IGF activity. Numerous tissue types express the IGF including brain, heart, lung, bone, placenta, and testes. Mature cartilage, developing cartilage, and synovial fluid exhibit the expression of IGF-1. Proliferation and differentiation induction in chondrocytes, MSCs, and embryonic limbs cell line in response of IGF-1 have been reported in different studies [75]. It is assumed that IGF-1 is involved in ECM anabolism through promoting type II collagen and proteoglycan synthesis [76]. Furthermore, IGF-1 suppresses the nitrogen oxide induced apoptosis and dedifferentiation in chondrocytes [75]. Primarily IGF-1 is pertinent to cartilage repairing, while IGF-2 is found to have an important role during fetal and embryonic developments. Isoforms of both IGF-1 and IGF-2 are activated through tyrosine kinase receptor (IGF-1R) [77]. IGF-1 binding with IGF-1R results in phosphorylation of various substrates which are involved in different important pathways such as Ras-ERK pathway and PI3K-phosphoinositide-dependent kinase 1-Akt pathways [78]. Growth retardation and organogenesis defects have been seen in mouse model with IGF-1 mutations during embryonic development. Osteoblasts, osteoclasts, and chondrocytes express IGF-1 and IGF-1R [77]. IGF-1 is one of the critically important mediators involved in the maintenance of the cartilage homeostasis through improving proliferation, chondrocyte survival, and proteoglycan synthesis [79]. IGF-1 induced chondrocyte migration in cartilage defected horse model; it also further improves the tissue repairing consistency upon combined use with chondrocytes [77].

### 3.4. Wingless Factors (Wnt) 

More than 20 Wnt members are found in vertebrates having a distinctive role in development. The Wnt binds with Frizzled receptors and subsequently cooperates with low density lipoprotein-receptor-related protein 5 and low density lipoprotein-receptor-related protein 6 [80]. Mostly Wnt activates canonical b-catenin-dependent signalling. Glycogen-synthase kinase 3b and casein kinase 1a phosphorylate b-catenin in absence of Wnt and consequently proteases degrade the b-catenin. Wnt regulates the expression of various genes. A Wnt member activates at least three different b-catenin-independent pathways such as activation of protein kinase C, PLC, calcium/calmodulin dependent protein kinase II, and JNK pathway [81]. Different Wnt members have important functions in the chondrogenesis and skeletal development, among which Wnt-1, Wnt-4, Wnt-7a, and Wnt-8 prevent chondrogenic differentiation, although exhibiting diverse effects during hypertrophy, whereas Wnt-5a, Wnt-5b, and Wnt-11 regulate proliferation and hypertrophic maturation of chondrocytes in growth plates during development [82]. The canonical Wnt signalling inhibits chondrogenesis and induces ossification. Wnt-3a exhibits controversial results in chondrogenesis [83]. In adult tissues chondrogenesis and osteogenesis processes require b-catenin [84]. In general, it appears that the Wnt playing important function in cartilage as chondrocytes development regulation and deregulation of Wnt networking could result in arthritis, specifically osteoarthritis.

## 4. Cell Culturing Methodologies and Chondrocytes Cellular Model Systems

Various culturing methods have been established to evaluate the effect of environment and culture conditions on specific normal chondrocytes and chondrocyte cell lines, being used as model systems for biological studies. Initially culturing of chondrocytes was developed during the 1960s for investigation of molecular and cellular biology of these cells [85]. Such efforts result in the development of various* in vivo* and* in vitro* culture models to study of chondrocyte biology, while suitability of a specific chondrocytes culture system purely depends on the nature of scientific question to be addressed. Recently, tissue engineering approaches are considered to treat the cartilage defects by using chondrocytes and suitable tissue scaffolds which can be carbohydrate based (agarose, alginate, chitosan, and hyaluronan), protein based (gelatin, collagen, and fibrin), or formed by hydrogels. Monolayer, three-dimensional culture method, pellet culture of chondrocyte, organ culture of cartilage slices, bioreactor culture, and implant models for tissue engineering have been reported.

Numerous cell systems have been applied to investigate the mechanism based on chondrocytes. Each model has its own unique properties which help in understanding the differentiation, proliferation, ECM maintenance, gene expression, pathophysiological effects, and ultimately cartilage formation. Generally employed chondrocyte models are the primary chondrocytes, immortalized cell lines, clonal cell lines, growth plate culture, and organ culture of cartilage slices.

### 4.1. Primary Chondrocyte Culture

Cells directly isolated from the living tissue are known as primary cells. These cells are directly received from source (human or animal) and started culturing. Primary culture is the* in vitro* establishment of the cell growth. These methods are capable of limiting the number of cell divisions. Usually, chondrocytes are obtained from animal model such as rabbit for research purpose. The chondrocytes are also obtained from the patients undergoing surgery for arthritis used to investigate the molecular mechanism of the diseases. Human chondrocytes are effectively grown. However, positive correlations have been found between donor age and primary confluence of cells [86]. Primary cell culture is the outstanding model for the study of physiological response and the mechanism behind joint diseases. Slow proliferation, dedifferentiation and insufficient amount of human chondrocytes are somehow notable interferences for using human originated primary chondrocytes.

### 4.2. Normal Clonal Cell Lines

Various life spans of the clonal nontransformed cells remain genetically similar during extended serial passage. These cells are used for the production of a sufficient number of cells. Establishment of different cell lines has been reported such as the HCS-2/8 clonal cell line having been established from chondrosarcoma of proximal humerus [87]. Extensive characterization of the cell line has been performed such as synthesis of collagen (types II, IX, and XI), proteoglycans, phenotypic variation, response against FGF, TGF-b, tumor necrosis factor- (TNF-) a, connective tissue growth factor, and cytokines [88]. The HCS-2/8 cells are capable of maintaining phenotypic markers of chondrocytes up to three years. These features make these cells a significantly better cellular model for undergoing mechanism of differentiation, gene expression, and cytokine stimulation response. Meanwhile, the origin of chondrosarcoma might affect the biological features.

The establishment of another human chondrosarcoma cell line (Ch-1) for etiology examination and cartilage-specific gene regulation has been reported [89]. Numerous mRNAs such as type XI collagen, aggrecan, proteoglycans decorin, TGF-b1 and P53, and tumor-suppressor genes were perceived in the Ch-1 cells, whereas type I and II collagen coding mRNAs were not identified [89]. Two other main proteins involved in chondrogenesis were also observed. The first is a CD-RAP, a secreted molecule restricted under normal conditions to differentiated chondrocytes and cartilage and Cart-1 (a homeobox) protein contributing to cartilage differentiation process [89]. These cells could be used to examine gene expression and etiology, but instability and mutations are major limitations for chondrocyte study.

ATDC5 is a prechondrogenic stem cell line which was isolated from AT805 embryonal carcinoma cells. During* in vivo* endochondral bone formation differentiation mechanism and stages are reproduced in ATDC5 cell line displaying the chondrocyte differentiation stages [90]. ATDC5 retain their chondrogenic progenitor cell properties in the presence of the insulin when grown in monolayer culture. These properties are shown during the initial stages of differentiation and upon reaching the condensation stage, ATDC5 cells form cartilage nodule [91]. Chondrocytes continue to grow in the cartilage nodules for about two weeks and cease their proliferation after the third week in cultures. Thereafter, hypertrophy occurs in association with the increase in alkaline phosphate activity and type X collagen expression, which are the key features of the endochondral bone formation [91]. Further, after two-week growth in the absence of the b-glycerophosphate supplements, mineralization can be observed in the cells [91]. The ATDC5 are initial cell model which exhibits entire spectrum of differentiation and henceforth could be used as* in vitro* model to study the mineralization through endochondral ossification.

CFK2 is clonal nontransformed fetal rat calvariae originated chondrocytic cell line. Extended monolayer culturing of these cells results in cartilaginous matrix deposition and formation of the focal cellular nodes. It has been reported that PTH (parathyroid hormone) and EGF (peptide regulatory factors) increase and retinoic acid and dexamethasone decrease the proliferation of CFK2 cells [92]. Retinoic acid and EGF inhibit focal nodes formation with decreased link protein expression and matrix deposition, whereas dexamethasone and PTH induce these effects [92]. The CFK2 cells serve as* in vitro* model for the study of the regulation of differentiation and cartilage matrix deposition in chondrocytes by different factors.

The rat calvaria originated cells RCJ3 (RCJ3.1C5.18) are clonal cell types showing the differentiation in large period monolayer culture method [93]. A number of factors influence the proliferation and differentiation processes. The retinoic acid and 1, 25 (OH)2-vitamin D3 suppressed the RCJ3 cell differentiation, while dexamethasone and low-contact matrix and dexamethasone induced the process. Amplified hormonal sensitivity for PTH and inhibited for prostaglandins (PG) can be found during differentiation. PGF2a increased and PGE1 inhibited the proliferation [94]. Increased mineralization and type X collagen expression studies have been reported in RCJ3 cells and these cells could be used for investigation of the growth plate chondrocytes differentiation [92].

### 4.3. Immortalized (Transformed) Clonal Cell Lines

The introduction of DNA into the cells causes immortalization and results in genetically modified cells known as transformed cell lines. Various cell lines including C-28/I2, T/C-28a4, and T/C-28a2 are established from primary chondrocytes using retroviral-mediated transfection approach with T antigen of Simian virus 49 [95]. These cells are commonly used in cartilage research and maintain their characteristic phenotype over 80 passages when grown in monolayer culture system [96]. Among these cell lines metabolic related gene expression pattern of C-28/I2 is closer to the primary chondrocytes [95]. The immortalized cells are capable of growing in monolayer culture without losing their morphology, although phenotypic variations have been found in these three cell lines. Another immortalized human chondrocyte cell line tsT/AC62 (temperature-sensitive) has been established and characterized to study arthritic related human chondrocyte functions and specific gene expressions [97, 98]. The specific transformed cells were produced by using a temperature-sensitive mutant of SV40-large T antigen (Tag) expressing retrovirus, which was used to transfect the primary adult articular chondrocytes and loss of Tag expression at 37–39°C (nonpermissive temperature) and expression of Tag 32°C (permissive temperature) were reported in temperature-sensitive established transformed cells (tsT/AC62 cells) [97]. Recently, immortalized (HPV-16 E6/E7) chondrocyte cell line is established through cationic lipofection (liposome-mediated) procedure by using 2 human papilloma virus type 16 (HPV16) early-function genes (E6 and E7) containing plasmid. These cell lines were produced to overcome trauma-damaged cartilage or osteoarthritic sources related potential problems [99]. Expression of key marker proteins such as II, IX, and X collagen is reported to be found in immortalized cell line up to 6 passages in both three-dimensional and serum-free medium cell culture systems, while articular diseased originated cells were found to downregulate type II collagen a cartilage-specific protein in monolayer culture. The described cells exhibited stabilized morphology and unrestricted proliferative potential. Furthermore, the use of normal articular cartilage is preferred to treat osteoarthritis or trauma-damaged cartilage [99].

## 5. Tissue Engineering and Chondrocyte Cell Based Therapies

**Figure 2:**
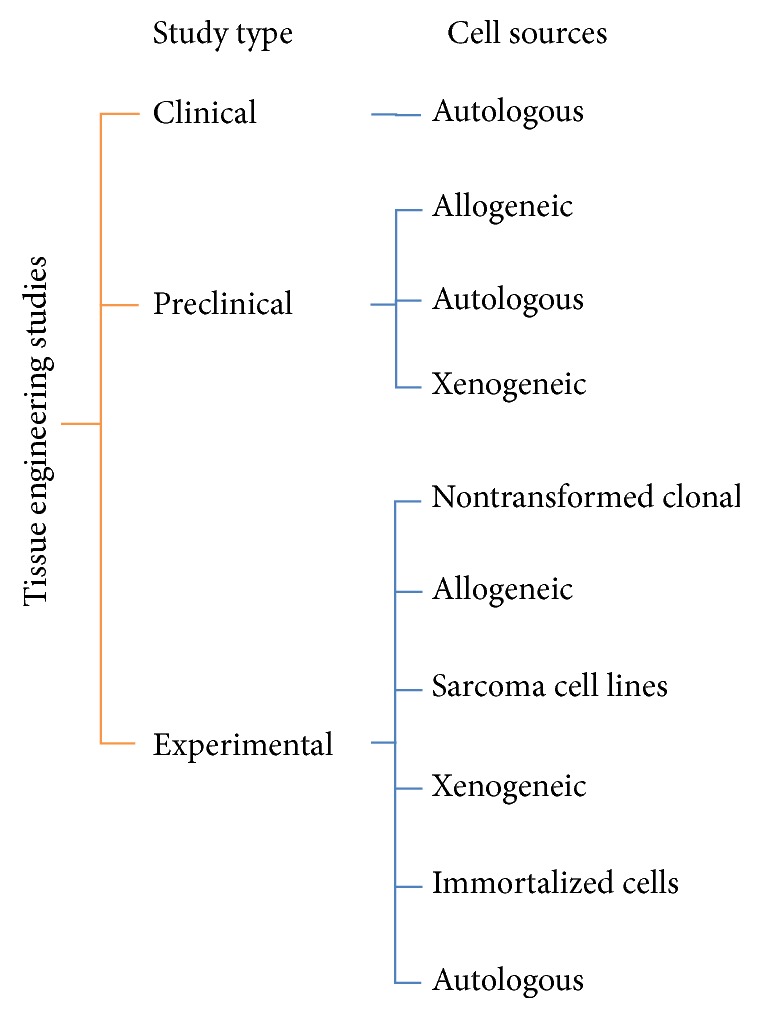
Various major cell sources used in tissue engineering studies.

Cell based therapy offers symptomatic relief, delay in disease progression, and long lasting repairing effects for cartilage regeneration. It is rapidly growing and developed therapy being applied for the cartilage repair [16]. Chondrocytes and mesenchymal stem cells are two focal cells targeted towards articular cartilage related pathologies. The autologous chondrocyte implantation is the most developed cellular based method to treat the cartilage ailments. There are numerous types of cell sources used in the tissue engineering and major cell sources are shown in Figure 2. Moreover cartilage and other cell lines such as immortalized cells as discussed earlier are being used to investigate the basic aspects of nonhuman environment.

### 5.1. Autologous Chondrocyte Implantation/Transplantation (ACI/ACT)

ACI is extensively used cell based technique for cartilage repair. Initially Swedish group put forwarded this method for tissue repair and more than 85% of patients got symptomatic relief in 2-year follow-up study [22]. Thereafter, in 1997 US Food and Drug Administration approved this technique. Three autologous chondrocyte implantation (ACI) generations have been developed and improved in the last two decades on the basis of several implantation/transplantation methodologies with the use of scaffolding material and synthetic membranes [100]. In first generation patient's specific periosteum is used as bioreactive chamber for cell growth and maturation [16]. The biomaterial-based membranes were used in the second generation to improve the limitations as periosteal delamination and hypertrophy. Comparatively, there is no use of periosteum or sutures in third generation, but chondrocytes are preseeded in three-dimensional scaffold material, implanted at defected site and fixed with specific substance such as fibrin glue. A brief description of each generation including cell source and cell delivery approach is presented in Table 2 [101].

The idea behind this method is to isolate the chondrocyte from same patients (autologous) grow them in in vitro conditions and use these cells to treat cartilage defect. ACI is a combinatorial approach involving surgical treatment and cell culture systems. Various modifications exist for ACI; for example, Brittberg [102] has reassessed the basic method and provides updated results. Initially, the cartilage tissue sample is taken from non-weight-bearing of defected cartilage area followed by transfer and storing in sterile conditions. Thereafter, cartilage biopsy is subjected to treatment with collagenase enzyme for the isolation of chondrocytes. The amplified number of chondrocytes in monolayer culture system fulfils the required number of cells for implantation for cartilage recovery. When cells are injected at defected site sewed with periosteal flap to prevent chondrocytes floating away from implanted side [22], periosteal flap not only supports the chondrocytes but also stimulates cartilage regeneration and possesses the chondrogenic capacities.

Initially the ACI method was applied on rabbit model for chondral defects; result showed that 82% of the defected cartilage area was covered with nascent cartilage [103]. Afterwards, chondrocytes, periosteal flap covering [104] or scaffold, and chondrocyte were applied in rabbit patella chondral defects [105]. Significantly, improved hyaline cartilage synthesis from 47 to 87% was observed in both cases even after one year. In further experiments chondrocytes were labelled with a fluorescent dye to confirm the function of the chondrocytes at implanted site through* in vivo* tracking. In another study persistence of the chondrocytes was observed at defected site in a goat model [106]. Comparative to rabbit model, there were not any substantial results observed in control and ACI treated canine model [107]. However, nascent hyaline cartilage was observed in more than 40% of defected area in canine model when treated with chondrocytes and scaffold; significantly higher result may be due to the use of scaffold material or could be synergistic effects [108]. This technique is globally applied on more than twelve thousand patients since 1987. Significant decrease in pain and cartilage-like tissue formation was shown in patient in response of ACI technique [109]. This method showed positive results in human patients, although repairing was not even in defected joint area [22, 109]. The efficacy of the microfracture and ACI techniques compared through randomised trial and both techniques showed satisfactory results in 77% of patients [110]. Clinical, radiographic findings in two treated groups and histological, clinical outcome results showed no significant differences and correlation, respectively. Long term continuation and follow-up were suggested to evaluate the disease progression and effectiveness of the methods [110]. Another randomised controlled trial showed comparable clinical findings after five years of treatments for characterized chondrocytes implantation (CCI) and microfracture (MF). Due to time importance in commencement of symptoms, it was proposed for future repairing and treatment strategies for knee cartilage [111]. Keeping in view of described facts of clinical trials there is still need of studies in human subjects and longer-term studies are suggested to measure ACI efficacy.

Even though encouraging clinical findings have been observed with use of ACI technique, still there are restrictions to be resolved. The principle limitations are associated with bioresponse of the periosteal flap, dedifferentiation of cells that results in loss of extracellular matrix related factors, a method used for expansion of isolated chondrocytes, and finally cost effectiveness and complexity of biopsy surgery [112]. Among all these complications related with ACI, periosteal flap detachment, hypertrophy, and delamination related clinical complications are often observed complications [113]. Usually scaffolds substances such as collagen sheets are used in second-generation ACI methods; collagen sheet along with chondrocytes has shown significant reduction in adverse effects such as hypertrophy. Due to holding capacity of scaffolds, these could be compromising agents for treatment of osteoarthritis patient missing cartilage rims. The third generation is also known as matrix induced autologous chondrocyte implantation/transplantation which is advantageous over classic ACI due to decreased surgical time and reduces fixation invasion and cell maintenance conditions. A limited number of studies have reported the tissue-engineered cartilage-like constructs for nonorthopedic application. However, the encouraging* in vitro* results have been observed, but there is still a need of* in vivo* validation.

## 6. Clinical Applications

**Figure 3:**
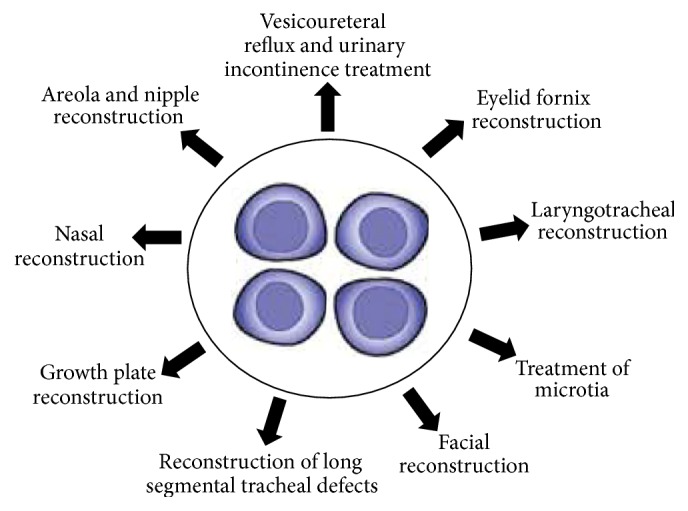
Application potential of the chondrocyte in tissue engineering based application.

Repair of cartilage tissue is an unresolved clinical issue. Despite numerous research studies conducted in this area still there is an insignificant development observed during the last decade. Articular cartilage degradation occurs in the course of pathological conditions in osteoarthritis or rheumatoid arthritis. It may also be induced in traumatic conditions such as occupational accidents or sports injuries [13]. Effective therapies are needed for improving the intrinsic repair mechanism of articular cartilage. In this regard, basic and applied experimental research has developed. It can bring the novel concept for therapy such as identification of new components and tissues involved in the pathological processes and the development of cell-, gene-, and tissue-engineered-based approaches that may positively influence the protective and repairing activities of this highly focused tissue. Clinicians and researchers are striving for a better understanding of cartilage healing process in order to develop more reliable methods of AC repair. Herein, application of chondrocytes for the treatment of cartilage related pathologies and healing nonorthopedic defects is described and shown in Figure 3.

### 6.1. Growth Plate Reconstruction

Inflammation, trauma, or fracture causes long bone growth arrest and bone invasion across the cartilage. A number of attempts have been made to treat these defects by using autologous chondrocytes, bone wax, fat tissue, and other substances [114]. One of the recent studies reported the growth arrest restoration in a sheep model upon using transplantation of chondrocytes grown in collagen gel [115]. Restoration of physeal arrest was found lower than 20% and not considered promising. Comparatively chondrocytes are persuasive cell for ECM synthesis. In another report, allogeneic chondrocytes along with agarose alleviated 50% of physeal arrest and long bone discrepancy in a rabbit model [116]. However, the use of allogeneic chondrocytes is controversial due to presentation of antigenic pathogens in patients and agarose use in human patients is not approved by relevant regulatory authorities. In atelocollagen gel culturing of autogenous chondrocytes has been reported and use of atelocollagen gel is advantageous due to lower immunogenicity [114]. Moreover, atelocollagen and chondrocytes preclude the ossification, length discrepancy, and angular deformity in a rabbit model.

### 6.2. Laryngotracheoplasty or Laryngotracheal Reconstruction

Laryngotracheoplasty method involves the use of cartilage interpositional grafting to treat stenotic airway, usually in the subglottic area of laryngotracheal defected patients. Mechanical integrity and stiffness features of chondrocytes obtained from articular, auricular, and nasal cartilage have been investigated to evaluate their biomechanical testing, among which auricular originated chondrocytes were found to have expected histological characteristics compared to the cells generated from other sources [117].* In vivo* experiments of autologous cartilage uses have shown significant results without cartilage degradation and exhibiting any side effects, whereas contrasting results of such degradation of implanted neocartilage and side reaction have been observed with the use of tissue-engineered material [118]. Studies on animal model are being performed to overcome the concern of body reaction; such studies include use of auricular chondrocytes.

### 6.3. Facial Reconstruction

Chondrocytes could be used to treat nonorthopedic conditions. Neocartilage fabrication could be used in predetermined shapes using tissue engineering technology and it proved the opportunity as patient-specific shaping for facial reconstruction. Recently, synthesis of human chondrocytes and polyglycolic acid nonwoven mesh construct has been reported [119]. Furthermore tubes and sheets of neocartilage were produced from construct and transplanted in animal models. In the same way molding of chondrocyte/alginate was used for cartilage in specific shapes such as three-dimensional tolerant chin and nose bridge structures [120]. Specific shapes having higher degree of precision and unique geometries are required in reconstruction of neck and head anatomical structures. Chondrocytes complex structures could serve as a substitute for original template and successful implantation has been reported in animal models [121].

### 6.4. Eyelid Fornix Reconstruction

Anophthalmos is a condition in which patients have small or absent orbit or have no visible ocular tissues; this condition can be acquired or congenital. Congenital anophthalmos is rare, while acquired condition is common, usually occurring after trauma but commonly by surgical enucleation. Several pathological conditions which cannot be recovered are the indication of enucleation such as painful blind eyes, phthisical or buphthalmic eyes, and malignant intraorbital tumors. Numerous studies have been carried out to reconstruct eyelid, but still challenging conditions are there to be resolved, including production of supportive eyelid and deep fornix for artificial eye. Therefore accomplishing of lasting natural appearance and relaxing retention is necessary in patients having defected anophthalmic orbits. Previously, use of auricular cartilage grafting has been used in follow-up study to treat anophthalmic patients of different ages for eyelid malposition and inferior fornix retraction. More than 90% of patients attained successful correction [122]. Satisfactory results have been reported in anophthalmic patients treated with auricular cartilage graft for lower lid retraction [123]. The use of prefabricated flap comprises lateral femoral circumflex vessels and auricular cartilage in tumor patients undergoing loss of eye lid due to prolonged maxillectomy and this procedure could be more applicable and beneficial for patients with inadequate recipient vessels [124].

### 6.5. Ear Reconstruction for the Treatment of Microtia

Histological and morphological assessment of subcutaneous implantation of autologous chondrocytes cultured with biodegradable polymers in animal model shows remodelling of chondrocytes in auricle shape and production neocartilage [121]. Significant results showed the potential of using tissue-engineered auricle to treat the microtia patients.

### 6.6. Nasal Reconstruction

Due to the low rate of resorption and higher infection resistant autologous cartilage is valid tissue graft source for nasal reconstruction. Auricular concha offers a suitable substitute for nasal reconstruction in patients with nasal septum defects [125]. Augmentation of facial form to the defect shape and volume of bioengineered reconstructive material is challenging limitation for the treatment of nose deformity. New cartilage was synthesised by using specific tissue and elastic nature of formed tissue was validated from biopsy specimen observation [126]. One of the previous studies showed the biomedical potential of the autogenous conchal cartilage for dorsal augmentation of saddled nose. In comparison to the common layering techniques increased dorsal height has been observed with the use of the endonasal or external rhinoplasty methodology [127]. Although composite grafting including auricular chondrocutaneous grafting is important and offering valid tissues having a similar morphology for nasal reconstruction, it has restriction of limited blood supply [128].

### 6.7. Areola and Nipple Reconstruction

Mastectomy is frequently implemented method for breast cancer treatment. Reconstruction therapy is a useful technique for the patient undergoing mastectomy and offering similar shape, size, and colour along with the same symmetry to the other breast. Different procedures are being explored for the synthesis of the elastic cartilage for their probable biomedical application. Previously, Cao et al. [129] used different polymers for the synthesis of tissue-engineered pig cartilage. He compared the suitability of chondrocytes growth along with calcium alginate, pluronic F127 gel, or polyglycolic acid. Thereafter, these autologous complexes were either implanted or injected into pigs.* In vivo* results showed the cartilage formation and furthermore fibrocartilage with significant dispersed collagen was observed in the calcium alginate and polyglycolic acid produced tissue after 6 weeks. Subsequently, effective approach was carried out to synthesise human nipple-like shape by using chondrocytes seeded thermosensitive polymer in immunocompetent pigs. In the start, synthesised construct was used for the formation of human nipple and studied through injecting the cartilage construct in the ventral surface of pig model. As a result of the chondrocyte-pluronic hydrogel implantation, human nipple-areolar-like structure was observed after 10 weeks [130]. Cartilage offers valuable characteristics devoid of subcutaneous depression due to dermal base support [131].

### 6.8. Treatment of Long Segmental Tracheal Defects

Feasible applications of the chondrocyte/biosorbable material or biodegradable material for different defects including long segmental tracheal defects have been observed in animal model studies. Synthesis of cartilage-like architecture has been observed during growth of human tracheal cartilage originated chondrocytes in three-dimensional DegraPol matrix [132].* In vitro* culturing of these cells in DegraPol results in proliferation recaptured spherical phenotype and cartilage-like tissue formation in 6 to 8 weeks. Additionally, alginate-encapsulated autologous chondrocytes with polyglycolic acid complex exhibited the approximately 20-week survival in bridge tracheal defected rabbit model [133]. Tracheal cartilage/polymer constructs could be used for treating long tracheal defects.

### 6.9. Vesicoureteral Reflux and Urinary Incontinence Treatment

Typically a urethral sphincter defect is often reported in incapacitated patients or aged persons and these defects cause vesicoureteral reflux and urinary incontinence. Since last century substitute therapy of suburethral tissue has been developed [134], because of clinical risk underlying immobilization of bulking substances in the bladder neck through surgery. Various bulking materials have been inspected through introducing into the neck of bladder, among which Teflon paste (polytetrafluoroethylene) was used to treat urinary incontinence and later on stopped due to side effects such as granuloma formation [135]. In early 1990s, bovine collagen material (Contigen, C.R. Bard, Inc.) was standard approved substance as injection therapy [134]; it also remained useful for shorter period of time. Glutaraldehyde cross-linked bovine collagen is one of the most widely used bulking materials but could not prove being stable substance. Stability of calcium alginate gel/chondrocytes complex has been reported where alginate gel works as bulking material in the animal models, indicating its feasible potential bulking material for therapeutic use [136, 137]. Here alginate gel is degradable material and works as cell carrier. Nonmigratory, noncarcinogenic, nonantigenic, and biocompatible natures of the chondrocyte-alginate construct have been investigated. Furthermore, construct can be delivered endoscopically. It has been reported that in a clinical trial against 29 children suffering from vesicoureteral reflux they have been treated with autologous cultured chondrocytes/alginate gel and about 60% of patients were relieved of reflux at 3 months in single treatment [135]. Further, a lower number of studies have been carried out for tissue-engineered cartilage-like constructs to apply for the treatment of nonorthopedic ailments. Though encouraging* in vitro* results are there, still* in vivo* confirmation is required to validate the use for biomedical application.

## 7. Current Challenges and Future Perspectives of Chondrocyte-Based Engineering

Recently, cell based articular cartilage products are gaining more attention all around the world, which indicates that repairing procedure can be improved through cell based engineering approaches. The major challenges in chondrocyte-based cartilage engineering include selection of the cell source, dedifferentiation and expansion procedures, how long it would be suitable to expand the chondrocytes before implantation, the range of the chondrocyte quantities against lesion volume in triggering the instinctive-like cartilage tissue formation, and assessment of the quality of* ex vivo* produced cartilaginous tissue through suitable biomarker. Various strategies are being followed to overcome these challenges. For example, changing in the formulation of the expansion medium and minimization of passage number could be used to mitigate chondrocyte dedifferentiation. Various chemical stimuli such as collagen cross-linking promoting agents, growth factors, and catabolic enzymes could be used to improve the quality of the engineered cartilage constructs. In addition, fluid-induced shear, tension, and compression are the mechanical stimuli exhibiting similar response. Aforementioned strategies could be useful for improved production of cartilage construct and effective utilization of chondrocytes through tissue engineering approach.

## 8. Conclusion

In summary, chondrocytes are unique and exceptionally located in the cartilage tissue. These cells are involved in the maintenance of extracellular matrix. Chondrocytes are being used for a variety of medical and surgical applications. Chondrocyte cellular therapy is predominantly targeted towards principal cartilage defects. The investigation of the suitable cell source is yet to be explored whereas chondrocytes seem to be the ultimate solution, but facing inevitable complications including proliferation and dedifferentiation. However, more studies are needed to evaluate the detailed culture conditions or method to be used for well-defined phenotypes chondrogenic lineages or production of biomaterial substances. Various attempts have been made for clinical application of chondrocytes. Different important biomaterials have been produced from chondrocytes for construction of the tissue-engineered cartilage having promising application in many disciplines. Importance of chondrocytes application in clinical practice is perceived from the studies discussed in this paper. This review focuses on chondrocyte origin, factors, and challenges to accomplishing efficacious and consistent results further focusing on comprehensive investigation to authenticate the extensive clinical acceptance of chondrocyte use as cell based technique. From the above discussion it can be concluded that chondrocyte is the focal probable cell source for tissue engineering approaches to produce promising cartilaginous constructs to treat cartilage related and nonorthopedic defects.

## Figures and Tables

**Table 1:** Advantage and disadvantages associated with the cells sources used in tissue engineering for cartilage repair.

Cell type	Advantage	Disadvantage/limitations
(i) Chondrocytes	(i) Promising cell source for cartilage repair (ii) More abundant than progenitor cells (iii) No severe clinical safety issues have been associated with the ACI technique (iv) Restricted to chondrogenic lineage	(i) Donor-site morbidity caused by cartilage harvest (ii) Chondrocyte dedifferentiation during cellular expansion (iii) Limited cells available and multiple surgical procedures involved are the hurdles of using chondrocytes (iv) Requires autologous cartilage

(ii) Adult mesenchymal stem cells (MSCs) (a) Adipose tissue-derived MSCs (AD-MSCs) (b) Bone marrow-derived MSCs (BM-MSCs)	(i) Easily obtained from tissues such as adipose tissue, bone marrow, and synovial membrane (ii) These cells have higher chondrodifferentiation capacity and proliferation rate (iii) Resistant to senescence (iv) Autologous cartilage is not required to obtained these cells	(i) Potential risks of induction or stimulation of tumorigenesis, colonization of nontarget tissues, transmission of infection, use of human (allogeneic) or animal serum-derived agents during cell expansion (ii) Reduced potentiality with age and disease (iii) Production of fibrocartilage instead of hyaline cartilage in the lesion and of terminal differentiation with cell hypertrophy and mineralization leading to the replacement of cartilage by bone (iv) Not restricted to chondrogenic lineage (v) AD-MSCs possess limited chondrogenic potential

(iii) Induced pluripotent stem cells (iPSCs)	(i) iPSCs have showed promising result in cartilage repair (ii) Cell amount is not issue and can be stimulated to obtained require amount (iii) Autologous cartilage is not required to obtain these cells	(i) Chondrogenic efficacy of iPSCs (ii) *Ex vivo* purification of calls (iii) Genetic modification associated with reprogramming protocols (iv) Teratogenesis perspective and *in vivo* tissue malformations

(iv) Embryonic stem cells (ESCs)	(i) Coculture with mature chondrocytes stimulates ESC chondrogenesis	(i) Teratoma formation (ii) Host immunorejection for clinical transplant

**Table 2:** Differences of autologous transplantation/implantation generations.

	First generation	Second generation	Third generation
Description	Autologous chondrocyte suspension with periosteum	Autologous chondrocyte suspension with collagen membrane	Autologous chondrocyte suspension in biomaterials

Cells source/delivery procedure	Carticel, periosteum patch ChondroCelect, characterized chondrocyte implantation (expanded population of chondrocytes that expresses a marker profile predictive of the capacity to form stable hyaline-like cartilage *in vivo*)	Chondro-Gide Bilayer membrane	MACI®, CaReS®, Tissucol®, NeoCart type I/III collagen, fibrin glue, type I/III collagen bilayer, BioSeed® C, esterified derivative of hyaluronate Hyalograft® C, fibrin glue + polymer-based scaffold of polyglycolic/polylactic acid and polydioxanone collagen type I gel seeded with autologous chondrocyte directly after isolation

Adopted from Samsudin and Kamarul, 2015 [101].
